# Radioactive Iodine Therapy of Differentiated Thyroid Carcinoma: Redesigning the Paradigm

**DOI:** 10.4274/2017.26.suppl.08

**Published:** 2017-01-09

**Authors:** Stanley J. Goldsmith

**Affiliations:** 1 Weill Cornell Medical College, Clinic of Radiology; New York-Presbyterian Hospital, Clinic of Radiology; Weill Medical College of Cornell University, Departments of Radiology and Medicine, New York, USA

**Keywords:** radioactive iodine, differentiated thyroid carcinoma

## Abstract

Radioactive iodine therapy has evolved over the past 70 years from treatment of known metastatic thyroid carcinoma to include adjuvant use to decrease the incidence of recurrent disease and to ablation of normal remnant tissue following thyroidectomy, even for minimal tumor involvement. Advances in laboratory testing, development of drugs useful in radioiodine treatment, as well as advances in radiation detection and imaging instrumentation, have progressively improved the utility of radioiodine therapy of differentiated thyroid carcinoma. Guidelines have proliferated and they have become more detailed and complex. This trend is likely to continue as the science and technology involved increases in sophistication and efficacy.

## Abstract (tr)

Son 70 yıl içinde radyoaktif iyot tedavisi metastatik tiroid kanseri tedavisinde kullanımdan minimal tümör tutulumunda bile nüks insidansını azaltmak ve tiroidektomi sonrasında kalan normal dokunun ablasyonunu sağlamak için adjuvan amaçlı kullanılmaya başlamıştır. Laboratuvar testlerindeki ilerlemeler, radyoaktif iyot tedavisinde yararlı ilaçların geliştirilmesi ve radyasyon algılama ve görüntüleme cihazlarındaki gelişmeler diferansiye tiroid kanserinde radyoaktif iyot tedavisinin yararını arttırmıştır. Bu konudaki kılavuz sayısı artmış ve daha detaylı ve karmaşık hale gelmiştir. Bu eğilim ilgili bilim ve teknoloji alanlarında kapsam ve etkinliğin artması ile birlikte gelecekte de devam edecektir.

## INTRODUCTION

Following the initial production of radioactive isotopes of iodine in 1937, physicians and physicists from the Massachusetts General Hospital and the Massachusetts Institute of Technology used these tracers, initially in animals, for the study of iodine physiology. In their initial publication based on studies in 48 rabbits, they concluded that “it is therefore logical to suppose that when strongly active materials are available, the concentrating power of hyperplastic and neoplastic thyroid for radioactive iodine may be of clinical or therapeutic significance”. Subsequently, the Massachusetts team studied the biodistribution and kinetics of iodine radiotracers in humans, particularly in patients with hyperfunctioning thyroid glands. They observed increased accumulation of the administered radioiodine in the thyroids of animals and patients with increased thyroid gland function (1). Shortly thereafter, in March 1941, Saul Hertz, MD, the Chief of the Endocrine Clinic at the Massachusetts General Hospital, and Arthur Roberts, a physicist at the Massachusetts Institute of Technology, administered multiple doses of radioactive iodine to a patient who had a hyper-functioning thyroid gland. The radioiodine was likely a mixture of the 12-hour half-life Iodine-130 and the 8 day half-life ^131^iodine (^13^1I). Their initial results on the treatment of hyperthyroidism with radioactive iodine, the first application of targeted radionuclide therapy, were presented at a scientific meeting in Atlantic City in 1942 but were not published until after the conclusion of World War II (2). Nevertheless, the news spread that radioactive iodine, in sufficient quantity, could retard thyroid function. In 1942, Samuel Seidlin, the Chief of Endocrinology at the Montefiore Hospital in the Bronx, New York began to evaluate the distribution of radioiodine in a patient who was exhibiting features of hyperthyroidism despite previously having had his thyroid gland removed for the diagnosis of thyroid carcinoma. Seidlin found no localization in the region of the thyroid but definite evidence of radioactivity accumulation in the right parietal region of the patient’s skull at the site of a soft tissue swelling that was demonstrated on subsequent x-ray examination to coincide with a lytic osseous lesion. He concluded that this mass was metastatic differentiated thyroid carcinoma. Following repeated administration of increased amounts of radioactive iodine preparations, less and less localization of radioactivity was noted in the sites of previous accumulation and the patient’s clinical symptoms of hyperthyroidism cleared with a notable gain in body weight and an overall improvement in his quality of life (2). At the time, this experience was unusual as other patients with malignant thyroid tumors did not demonstrate vigorous radioiodine uptake. It was also in 1942 that Albert Keston and colleagues at the College of Physicians and Surgeons of Columbia University observed vigorous radioiodine uptake in boney metastases following at least sub-total thyroidectomy, leading to the conclusion that metastatic differentiated thyroid carcinoma concentrated radioiodine better if the bulk of the thyroid gland had been removed previously (3). Following the conclusion of World War II, the embargo on publication of research on nuclear materials was lifted and medical reports of these experiences with radioactive iodine as a therapeutic agent were published. Thus, targeted radionuclide therapy was born. The term “atomic cocktail” was coined to describe treatment of thyroid disease (both thyroid cancer and hyperthyroidism). The “Atoms for Peace” program was initiated, stimulating public interest and government support for the production of radionuclides for use in medicine.

## A PARADIGM DEVELOPS

It was soon realized, however, that treatment of thyroid cancer was not as simple as simply administering radioactive iodine orally or parenterally.

- There was the issue of patient preparation. In 1948, Seidlin reported his several year experiences with the use of radioiodine in patients with differentiated thyroid malignancy, confirming Keston’s findings and concluding that it was necessary to eliminate the normal thyroid tissue to optimize radioiodine uptake by thyroid metastases. This recommendation was reaffirmed by Drs. William Blahd and William Bierwaltes, clinical thyroidologists in Los Angeles and Ann Arbor (4,5). In 1984, Bierwaltes concluded “there is no question… we should ablate normal thyroid as part of the treatment of differentiated thyroid carcinoma” (5).

- Over time, the protocol involving patient preparation prior to ^131^I therapy became more complex. After 1947, the radionuclide used was likely always ^131^I. It was recognized that ^131^I uptake of remnant tissue and differentiated tumor was augmented when in addition to removal of the bulk of the normal thyroid tissue, ^131^I uptake of residual tissue and tumor was stimulated when the patient was hypothyroid; that is deprived of thyroid hormone producing elevation of serum throid-stimulating hormone (TSH)-although this assay was not routinely available for many years. The practice arose to discontinue replacement thyroid hormone-usually desiccated thyroid hormone, prepared from bovine or porcine thyroid tissue that had became available from the meat industry. Subsequently, animal-origin TSH also became available by extracting TSH from the pituitary gland of slaughtered animals. Although the use of animal-derived TSH was more convenient than discontinuing thyroid hormone replacement and allowing hypothyroidism to develop over 4-6 weeks, the practice was discontinued in the early 1970s because of urticarial and other allergic reactions to the foreign protein. Because the TSH assay was not as conveniently available as it is today, there was a delay in obtaining TSH levels. Nevertheless, the practitioner had the option of determining that there was clinical evidence of hypothyroidism. It was considered good practice to at least draw a blood sample to retrospectively confirm that the serum TSH was indeed elevated to 30 uU/ml or more.

- The practice of radioiodine therapy of differentiated thyroid carcinoma was becoming more complex. The patient was initially evaluated by the clinician and advised to discontinue thyroid hormone. After 3-4 weeks, a low iodine diet was initiated and at week 5 or 6, the patient was evaluated clinically for evidence of a thyroid deficient state, followed by administration of a dose of ^131^I either to assess if there was evidence of functioning metastases, or as ^131^I therapy.

- Until the development of mechanical scanning equipment, the involved clinician performed “hand scanning” several days after the ablative or therapy ^131^I dose, or at follow-up evaluations; that is, the clinician held a radiation detecting instrument over various body parts, particularly the neck, anterior mediastinum, spine and the long bones. The instrumentation evolved from simple Geiger-Muller type devices to shielded probes with scintillation crystals and a photomultiplier tube. With sufficient shielding, the device provided more specific localization of the radioiodine accumulation. The clinician relied on an audio output proportional to the count rate. Hand-scanning was supplemented by collection of the entire urinary output for 24-48 hours. An aliquot of the total volume was counted and the percent of the total excretion of the administered radioactivity was determined. If all of the activity could be accounted for based on counting an aliquot of the collected urine, the patient was deemed to be free of functioning metastases. Of course, the urine collections were rarely complete, necessitating a decision by the clinician whether or not the discrepancy represented simply incomplete collection or retention by tissue metastases which could be confirmed by the radiation detecting probe. Although the hand-held probe and lead shielding weighed perhaps 25-30 lbs, this practice continued through the 1960s when it was replaced by rectilinear scanners that produced life size images of the scanned area (6).

- In the United States, the practice developed that requiring hospitalization and relative isolation of patients receiving >30 mCi of ^131^I until external detecting instruments indicated that the whole body burden had decreased below that level.

- Subsequently, Mazzaferri (7) reported on the clinical outcome of 1,004 patients with differentiated thyroid carcinoma who were more than 45 years old and had tumors greater than 1.5 cm, either given no post-operative medical therapy, received thyroid hormone replacement or remnant ablation with 1.1-7.2 GBq (30-200 mCi) of ^131^I. Median follow-up was 21.3 years, 18.7 years and 14.7 years, respectively. There was no difference identified between patients receiving a low or high dose of ^131^I. In the group who received ^131^I ablation, regardless of the dose, tumor recurrence was significantly lower, fewer patients developed distant metastases and there were fewer cancer deaths than in the other groups.

- Hence, the practice of radioactive iodine therapy as treatment for metastatic disease evolved toward a paradigm that advised radioactive iodine (^131^I) for virtually all patients with the diagnosis of differentiated thyroid cancer (DTC) to include treating patients with:

- Metastatic disease (the original indication for radioiodine therapy), treatment of patients with probable residual tumor based on histopathology evidence of positive margins, extra-thyroidal extension or tumor involvement in resected lymph nodes,

- Adjuvant therapy based on the assumption that there might be occult tumor based on assessment of risk factors,

- Ablation of remnant tissue to improve the sensitivity of radioactive iodine to detect and destroy recurrent disease. As a practical matter, there is little difference between adjuvant therapy (radioiodine administration based on the assumption that there is residual disease even though it has not been confirmed) and ablative therapy, radioiodine administration to eliminate residual normal tissue so that it would not compete for administered radioiodine in the event that there was tumor recurrence although, in general, practitioners often administered 30 mCi doses (1.11 GBq) as an ablative dose whereas 75-150 mCi (2.75-5.50 GBq) doses were recommended for adjuvant therapy (8,9).

## EXISTING PARADIGMS

The present practice of radioiodine therapy including ablation has grown increasingly complex. Professional and scientific organizations responded to these complexities by the creation and publication of Guidelines setting forth a “paradigm” for the utilization of 131I in the management of patients with differentiated carcinoma arising from the follicular cells of the thyroid. Paradigms are generally assumed to provide useful guidance for patient management of complex clinical situations (Boxed Text 1).

Definition of “Paradigm”

In the on-line Urban Dictionary, there are multiple definitions of the word “paradigm”:

1. “An example the majority of people follow; an established set of values or ways” (Miso)

2. “… a closed set of scientific theories that is coherent and is well accepted by the larger scientific community” (Nathan Leichoz)

3. “The most annoying and misused word in the English language; used intentionally by stupid people to sound smart or by smart people to sound unintentionally stupid” (Jambone)

In 2002, the Society of Nuclear Medicine published Version 2 of a Guideline entitled “Procedure Guideline for Therapy of Thyroid Disease with 131I“ (Boxed Text 2) (9).

Version 2 of the Society of Nuclear Medicine Guideline in the use of ^131^I for the Treatment of Thyroid Disease

Part I: Purpose

Part II: Background information and definitions

Part III: Common indications

Part IV: Procedure

Part V: Issues requiring further clarification

Part VI: Concise bibliography

Part VII: Disclaimer

This document provided clinical practice guidelines for the use of ^131^I for the treatment of hyperthyroid, non-toxic goiter and thyroid carcinoma. The document was 5 pages in length as published in the Journal of Nuclear Medicine. The Guideline consisted of 6 parts (Boxed Text 2). Part V of this Procedure Guideline is entitled “Issues Requiring Further Clarification” and identifies the following:

- The use of ^131^I whole-body imaging before ^131^I therapy for thyroid cancer and whether “stunning” of the thyroid remnant occurs.

- The role of alternative imaging agents such as ^123^I to avoid stunning.

- The necessity of treating small (<1.0 cm) papillary cancers with ^131^I.

- Treatment of ^131^I-scan-negative, thyroglobulin positive patients.

- The role of recombinant human TSH in therapy.

Part VI is a Concise Bibliography containing 15 references. The Guideline concludes with a Part VII: Disclaimer which is 1 paragraph in length concluding with the following sentence: “Advances in medicine occur at a rapid rate. The date of a guideline should always be considered in determining its current applicability.” In 2012, Version 3 entitled “The Society of Nuclear Medicine Practice Guideline for Therapy of Thyroid Disease with ^131^I 3.0” was published; it is 19 pages in length (Boxed Text 3) (10).

Version 3 of “The Society of Nuclear Medicine Practice Guideline for Therapy of Thyroid Disease with ^131^I 3.0”

Preamble

Introduction: patient management, licensure

Goals

Definitions: risk levels

Common clinical indications

Qualifications and responsibilities of personnel (in the United States)

Procedure/specifications of the examination

Documentation/reporting

Equipment specification

Quality control and improvements

Safety, infection control, and patient education concerns

Radiation dosimetry

Acknowledgements

References

Approval

The portion specific to ^131^I therapy of thyroid cancer alone is 8 pages (Boxed Text 4).

“The Society of Nuclear Medicine Practice Guideline for Therapy of Thyroid Disease with ^131^I 3.0”

VI. Procedure/specifications of the examination

A. Therapy of Graves disease, toxic nodules, and nontoxic nodular goiter

B. ^131^I therapy of thyroid cancer to ablate post-thyroidectomy remnants and destroy residual or recurrent tumor

1. Indications for treatment with ^131^I: relationship to staging

2. Patient preparation and information the patient needs: diet, thyroid-stimulating hormone level, informed consent, side effects

3. Information required by the physician performing the procedure: blood tests

4. Selection of activity

5. Therapeutic procedure for administration of ^131^I

6. Follow-up

C. Radiation Safety issues, patient discharge, home instructions

D. Interactions of ^131^I with other forms of diagnosis or treatment

E. Radiopharmaceuticals

F. Issues requiring further clarification

Within this Section, there is a sub-section entitled “Issues requiring further clarification”.

1. Utility of routine use of ^123^I or ^131^I whole-body imaging, especially SPECT/CT in patients after total thyroidectomy before initial ^131^I ablation therapy for thyroid cancer,

2. Pathologic and prognostic significance of stunning of the thyroid remnant and metastatic deposits,

3. Diagnostic role of alternative imaging agents for thyroid cancer such as ^123^I, ^124^I and ^99m^Tc,

4. The role of ^124^I in thyroid dosimetry, and the efficacy of lesion dosimetric planning,

5. The necessity of therapy for low risk papillary cancers less than 1.0 cm in diameter if there is an unfavorable molecular assessment [e.g. BRAF expression (a proto-oncogene encoding a serine/threonine protein kinase called B-RAF)], unfavorable histology, and no evidence of distant metastases,

6. Equivalence between recombinant human TSH… and endogenous TSH elevation from thyroid hormone withdrawal,

7. Frequency and length of long term follow-up after ^131^I therapy… in a variety of clinical situations,

8. Prediction of the time required for the TSH to rise sufficiently… after thyroid hormone withdrawal… before ^131^I therapy,

9. The need to attain a serum TSH level of at least 30 uU/ml,

10. Standardization of ^131^I dosimetry to deliver… ablative radiation doses to remnants,

11. Benefits and risks of empiric high-activity ^131^I therapy (e.g. >9.25 GBq [250 mCi]) for patients with serum thyroglobulin elevation but negative iodine scintigraphy,

12. Therapeutic benefit of administered activities in excess of, for example 9.25 GBq in iodine-avid metastatic disease, relative to lower activities of ^131^I,

13. Determination of whether external-beam radiotherapy delivered to the neck metastases before therapeutic ^131^I decreases the subsequent ^131^I therapeutic effect,

14. Prevention of radiation sialadenitis and oral mucositis.

In 2008, the European Association of Nuclear Medicine (EANM) published a 19 page Guideline in the European Journal of Nuclear Medicine and Molecular Imaging (11). This guideline is quite complete. There are no substantial differences between it and the Society of Nuclear Medicine and Molecular Imaging (SNMMI) Guideline. The Abstract and abstracted Discussion of the publication reads as follows”.

“**Abstract:** The purpose of the present guidelines on the radioiodine therapy of DTC formulated by the EANM Therapy Committee is to provide advice to nuclear medicine clinicians and other members of the DTC-treating community on how to ablate thyroid remnant or treat inoperable advanced DTC or both employing large ^131^I activities. Discussion: For this purpose, recommendations have been formulated based on recent literature and expert opinion regarding the rationale, indications and contraindications for these procedures, as well as the radioiodine activities and the administration and patient preparation techniques to be used.“

The International Atomic Energy Agency has published a 271-page Technical Document entitled “Nuclear Medicine in Thyroid Cancer Management: A Practical Approach” that covers essentially the same material, providing greater detail in the description of procedures (12). There are still some differences in radiation safety requirements among several European countries. In this regard, the United States is currently less restrictive in terms of the need to isolate patients following ^131^I administration, basing the requirement of exposure of others and taking into account the term “occupancy factor”. Since, except for overnight sharing of a bed, most individuals will not spend an extended period in close proximity to another individual, the exposure that any member of the public or even immediate family would receive is only a fraction of the total radiation flux emitted from the treated individual. The exposure another individual would receive is a product of the residual activity in the treated patient (a value that in the case of ^131^I decreases rapidly based on renal excretion), the proximity to the patient as a source (with the radiation flux falling off rapidly based on the inverse square of the distance; as a practical matter, at one meter, the value is less than 10% of the flux detected at the source) and the duration of time at that location-a value that is reflected in the occupancy factor rather than assuming total exposure of another individual from all of the source activity. Other than that element of radioiodine administration, there is general agreement about the issues and the degree of certainty or uncertainty in the benefits associated with any component of the procedures and decisions involved.

Finally, in 2015, the American Thyroid Association (ATA) published in it’s original form as a 411-page document entitled “Management Guidelines for Adult Procedures with Thyroid Nodules and Differentiated Thyroid Cancer”. Seventy-two pages deal with the diagnosis, procedures and decisions involved in the assessment of thyroid nodules. Nevertheless, of the remaining 339 pages, 278 are devoted to various aspects of DTC and management. There are 94 pages of references and 1078 references are cited. This document has been reformatted and published in the journal Thyroid in January 2016 (13). The ATA Guidelines provide recommendations about what procedure or action is reasonable given the evidence available in addition to assessing the quality of the research or data upon which published conclusions are based (and providing a grade for the quality of that data). “As of February 2014, the SNMMI Guidelines will now be referred to as Procedure Standards. This change was initiated in an effort to better reflect the terminology being used by external organizations. Any previous Practice Guideline that describes how to perform a procedure is now considered as SNMMI Procedure Standard” (Quoted from the SNMMI website under the heading Procedure Standards). Collectively, these Guidelines are quite comprehensive. They present a thoughtful review of the state of knowledge at the time the Guideline was released and published. In addition, they identify areas that require further clarification (research). Nevertheless, the diagnosis and management of patients with differentiated thyroid carcinoma continues to be the subject of a great deal of clinical and when possible, basic research. Recently, in fact, a remarkable observation has been reported: the potential use of a class on pharmaceuticals, tyrosine kinase inhibitors, that suppress or counteract the genome defect that produced a proto-oncogene involved in the failure of differentiated thyroid carcinoma cells to express the Sodium Iodide transporter, thus restoring the ability of these cells to trap and organify (thus retaining) iodide ion, in general, and ^131^I in particular into metastatic sites that had not demonstrated radioiodine ^131^I uptake. The potential for this exciting therapeutic intervention was initially observed in a subset of patients in whom the potential for effective ^131^I therapy was predicted based on ^124^I positron emission tomography imaging and dosimetry which was subsequently confirmed (14).

## REDESIGNING THE PARADIGM

This review has identified existing Guidelines or Procedure Standards (the current preferred nomenclature) for Radioactive Iodine Therapy of Differentiated Thyroid Carcinoma. They are comprehensive and lengthy. Upgrades will be necessary from time to time as new basic research identifies opportunities for clinical innovations and clinical research validates opinions and recommendations of the “cognoscenti”. At this time, however, other than enriching what already exists with appropriate upgrades, there does not appear to be a need to “redesign the paradigm”.
